# Effects of Radio Frequency Heating on the Stability and Antioxidant Properties of Rice Bran

**DOI:** 10.3390/foods10040810

**Published:** 2021-04-09

**Authors:** Yen-Hui Chen, Yu-Fen Yen, Su-Der Chen

**Affiliations:** Department of Food Science, National Ilan University, Number 1, Section 1, Shen-Lung Road, Ilan City 26041, Taiwan; yenhui@niu.edu.tw (Y.-H.C.); abcz550068@gmail.com (Y.-F.Y.)

**Keywords:** radio frequency (RF), rice bran, lipase, antioxidant, stabilization

## Abstract

Radio frequency (RF) technology is considered as a rapid heating method. Lipase in rice bran could highly accelerate lipid oxidation. The objectives of this study were to establish the radio frequency heating conditions for lipase inactivation and to evaluate the stability and antioxidant capacity. The results showed that the suitable electrode gap for a 1 kg sample load was 6 cm, and it only took 2 min to heat rice bran from 25 °C to 100 °C. Besides, there were no significant differences in the total phenolic content, flavonoid content and color between the untreated and RF-treated group, and the DPPH free radical scavenging activity of the RF treatment reached 84.8%. The acid value, free fatty acid content and peroxide value of the RF-treated rice bran met the quality standard after 8 weeks of storage at 4, 25 and 37 °C. In summary, this study provides valuable information about the RF heating procedure, and shows the great potential of RF technology for stabilizing rice bran efficiently.

## 1. Introduction

Rice bran is a byproduct from the rice milling process, and it is about 7–8.5% of milled rice. Moreover, rice bran is rich in proteins (~12.32%), lipids (~20.31%), digestible carbohydrate (~12.12%) and dietary fiber (~28.6%). In addition, rice bran is a potential source of natural antioxidants, such as γ-oryzanol, tocopherols, tocotrienols and polyphenols [1,2,3]. Color measurement could be used as an indicator for evaluating the treatment conditions. Due to the Maillard browning reaction, a higher temperature and longer heating time would cause a lower L value of the heat-treated rice bran [4]. Although rice bran is mainly used as animal feed due to its nutrient content and low price, it could be produced as an edible food ingredient by employing suitable techniques. However, the major restrictive cause for the application of rice bran is its instability during storage, which is largely due to enzymatic activity, including lipase and lipoxygenase [5]. The activity of these enzymes is responsible for the hydrolysis of triglycerides, which cause deterioration of rice bran. For human consumption, the free fatty acid content should not exceed 5% [6], but the free fatty acid of rice bran reaches 5–7% within the first 24 h [7]. Therefore, developing an adequate process to stabilize rice barn immediately after the milling process is important for preventing deterioration.

Many stabilization techniques have been proposed, such as hot air at 120 °C for 30 min [8], steam treatment at 121 °C for 5 min [9], microwave heating in 2450 MHz for 2 min [10], infrared treatment at 40 °C for 2 h [8], ohmic heating [11,12,13] and extrusion treatment [14]. However, most of these treatments have some problems, such as a high temperature or long processing time, that could damage the nutrient content of rice bran. In addition, the disadvantages of these treatments are a small loading, less flexibility and high operating cost.

A radio frequency (RF) consists 1 to 300 MHz electromagnetic waves, and RF has been widely used in the food industry and pharmaceutical as a heating process [15,16]. The principles of RF and microwave heating are basically the same. By using the RF treatment, the electric field that is generated by the currents makes the ions of the sample to quickly migrate or water molecules to rapidly rotate, and the friction of water molecules could produce heat. However, compared to microwave heating, the major benefit of RF treatment is the deeper penetration depth and uniform heating effect in bulk goods [16].

The RF system for treating rice bran has been discussed in several studies, in which parameters such as the electrode gap, power and time were investigated. Rice bran (200 g) was treated by RF system (6 kW, 27.12 MHz) heating with an electrode gap of 10 cm for about 2.5~3 min at 100 °C, combined with holding it in hot air for 15 min to improve uniformity, to eventually decrease to 19.2% and 5.5% the lipase and lipoxygenase activities, respectively. The free fatty acid content and peroxide value of the RB oil extracted samples treated under these conditions remained below the acceptable limits, even following 60-day storage at 35 °C. [17]. The RF heating at a 100 to 120 °C holding time for 5 min was applied for the stabilizing rice bran, and results showed the structure of the protein isolates was significantly changed, which improved the absorption and emulsifying properties [18].

In a previous RF study, rice bran (400 g) was stabilized by a hot air-assisted RF heating system (12 kW, 27.12 MHz) at a low temperature of 100–105 °C for 15 min or at high temperature of 110–115 °C for 6 min without a significant effect on the protein, lipid, total tocotrienol, total tocopherol and fatty acid composition, but reducing the free phenolic content of the rice bran [19]. The dielectric properties (ε′ and ε″) of rice bran during RF heating were greatly influenced by the frequency, temperature and moisture content of the rice bran [20]. In addition, researchers have only selected 200 g [17] and 400 g [19] rice bran by applying 6 kW and 12 kW 27.12 MHz RF equipment, which still required 15 min and 6 min for rice stabilization, respectively.

In order to improve the thermal efficiency and larger loading for industrial application, the objectives of this study were (1) to establish the radio frequency (5 kW, 40.68 MHz) heating procedure for inactivating the lipase of 1 kg rice bran; and (2) to evaluate the stability and antioxidant capacity of rice bran after RF treatment in order to promote the application and the value of rice bran.

## 2. Materials and Methods

### 2.1. Materials and Rice Bran Package Preparation

Rice bran (Indica rice, Taichung No. 10) was provided by Wujie Township Farmers’ Association (Ilan, Taiwan), and the moisture content was about 14%. They were packed in 1 kg vacuum bags, and then stored at −18 °C to prevent hydrolysis of the fatty acids by lipase activity. The rice bran was packed in a 1 kg vacuum bag (length × width × height = 25 × 19 × 3.5 cm^3^). Due to the powdery form of the rice bran, the sample was packed in a 1 kg vacuum bag during the processing. The sample loading of 2 and 3 kg was done using 2 and 3 bags of the vacuum-packed rice bran, respectively.

### 2.2. RF Heating Procedure Establishment for Lipase Inactivation

#### 2.2.1. RF Power Output Measurement

The sample loadings were 1, 2 and 3 kg, and the electrode gaps were set from 6 to 16 cm, depending on the treatment conditions. The time of each treatment was 5 s. The current output (A) was measured tree times for calculating the power output of the RF instrument (5 kW, 40.68 MHz, Yh-Da Biotech Co. Ltd., Yilan, Taiwan). The maximum output current and power output were 1.6 A and 5 kW, respectively. The RF power output was calculated by the following equation: RF power output (kW) = (A/1.6) × 5.

#### 2.2.2. RF Heating Rate and Energy Consumption Measurement

To start, 1 kg of vacuum-packed rice bran was RF-treated for 30 s, and the electrode gaps were selected as 6, 8, 10 and 12 cm, respectively. The measurement of temperature was then performed in triplicate by an infrared thermometer. The current output (A) was measured, and the RF stabilization energy consumption was calculated by the following equation:

$$\mathrm{energy}\;\mathrm{consumption}\;\left(\mathrm{kWh}\right)\;=\;220\;\times \;A\;\times \;\sqrt{3}\;\times \;\mathrm{heating}\;\mathrm{time}\;\left(h\right)$$

### 2.3. Storage Studies of RF-Treated Rice Bran

Rice bran was heated by RF treatment for 2 min, and the electrode gap was 6 cm. Untreated and RF-treated rice bran were stored under 4, 25 and 37 °C for 1, 2, 3, 4 and 8 weeks for further analysis, namely, the acid value, free fatty acid content, peroxide value and DPPH assay. According to an accelerated laboratory test [15], the RF-treated rice bran was stored at 37 °C for 10 and 20 days, which simulated 1- and 2-year storage at 4 °C day.

### 2.4. Analytical Methods

#### 2.4.1. Lipase Activity Assay

A total of 2 g of rice bran sample was mixed with 10 mL of sterile water. After ultrasonic extraction for 20 min, the solution was centrifuged at 5500 rpm for 15 min, and then the supernatant was collected as the enzyme solution. Lipase activity assay was determined by the method as described by Rakkan et al. [21], with modification. The standard solution was prepared by p-nitrophenol. The 0.15 mL of enzyme solution was mixed with 1.025 mL of phosphate buffer and 0.025 mL of 5 mM nitrophenyl acetate solution. The reaction was carried out in a dry bath (DB200-2-110, SCI-MISTRY CO., Yilan, Taiwan) at 37 °C for 15 min. The absorbance was determined at 410 nm by a spectrophotometer (Model U-200l, Hitachi Co., Tokyo, Japan). All experiments were performed in triplicate. The lipase activity was calculated by following equation:

Lipase activity (U) = relative p-nitrophenol concentration × dilution factor × total volume

The lipase activity retention (%) of untreated rice bran was defined as 100%.

#### 2.4.2. Antioxidant Component and Activity Assay of RF Stabilized Rice Bran

##### Microwave Extraction of Rice Bran

Microwave extraction of rice bran followed Reference [5]: 2.5 g of sample was mixed with 25 mL of ethanol (solid to liquid ration = 1:20) under microwave extraction for 5 min. The solution was centrifuged, and the extract supernatant was collected for further antioxidant component and activity assay.

##### Total Phenolic Content and Flavonoid Content

The total phenolic content of the rice bran was determined by the Folin–Ciocalteu method, and the total flavonoid content was measured by the aluminum chloride colorimetric method [22]. For total phenolic content measurement, 1 mL of extract supernatant was mixed with 1 mL Folin–Ciocalteu phenol reagent and 0.8 mL of 7.5% Na_2_CO_3_. After 30 min in the dark room, the absorbance was determined at 765 nm by a spectrophotometer (Model U-200l, Hitachi Co., Tokyo, Japan). Gallic acid was chosen as a standard. For the flavonoid content assay, 1 mL of the extract supernatant was added into 1 mL of 2% methanolic (AlCl_3_·6H_2_O). After incubation at room temperature for 10 min, the absorbance was measured at 430 nm by a spectrophotometer (Model U-200l, Hitachi Co., Tokyo, Japan). Quercetin was chosen as a standard. All experiments were performed in four repetitions.

##### Reducing Power and DPPH Radical Scavenging Ability Assays

The reducing power and DPPH radical scavenging ability assays were done following Reference [23], with modification. For the reducing power assay, 2.5 mL of the extract supernatant was mixed 2.5 mL of 0.2 M phosphate buffer solution (KH_2_PO_4_) and 2.5 mL of 1% C_6_N_6_FeK_3_. After incubated in a hot water bath at 50 °C for 20 min and then cooled to room temperature, 2.5 mL of 10% trichloroacetic acid (TCA) solution was added. The solution was centrifuged at 3000 rpm for 10 min, and the supernatant was collected. Then, 5 mL of the supernatant was mixed with 5 mL of H_2_O and 1 mL of 0.1% ferric chloride (FeCl_3_). After reacted for 10 min, the absorbance (ABS) was measured at 700 nm by a spectrophotometer (Model U-200l, Hitachi Co., Tokyo, Japan). The 5 mg/mL ascorbic acid, BHA and EDTA were used as standards. Reducing power was calculated by the following equation:

Reducing power = ABS _sample_ − ABS _control_

For the DPPH assay, 2 mL of the extract supernatant was mixed with 2 mL of 0.2 mM 1, 1-diphenyl-2-trinitrophenylhydrazine (DPPH-MeOH) solution. After incubated at room temperature for 30 min, the absorbance (ABS) was measured at 517 nm by a spectrophotometer (Model U-200l, Hitachi Co., Tokyo, Japan). The 20 mg/mL ascorbic acid, BHA and EDTA were applied as standards. The scavenging effect (%) was calculated by the following equation:

Scavenging effect (%) = ((ABS _control_ − ABS _sample_)/(ABS _control_)) × 100%

All experiments were performed in four repetitions.

#### 2.4.3. Color Measurement

The color of the sample was measured with a Color Difference Meter (JP7200F, Juki Co. Ltd., Tokyo, Japan) and standardized against a calibration white plate (X = 82.48, Y = 84.23, Z = 99.61; L * = 92.93, a * = −1.26, b * = 1.17). The parameters determined were degrees of lightness (L *), redness (+a *) or greenness (−a *), and yellowness (+b *) or blueness (−b *). All experiments were performed in six repetitions.

#### 2.4.4. Acid Value and Free Fatty Acid Content

The acid value was determined by the Chinese National Standard (CNS 3647 N6082). A total of 5 g of sample was added into 30 mL of the mixture of ethanol and ether for ultrasonic extraction for 20 min. After centrifugation, 10 mL supernatant was collected. Then, 2–3 drops of phenolphthalein indicator were added into the supernatant. A 0.01 N potassium hydroxide was used for titration. All experiments were performed in four repetitions.

#### 2.4.5. Peroxide Values

The peroxide values were analyzed as per the method of the Chinese National Standard (CNS 3650-N6085). A total of 5 g of sample was mixed with 18 mL of acetic acid and 12 mL of isooctane, and then added 0.5 mL of saturated potassium iodide and 30 mL of distilled water. A 0.1 N sodium thiosulfate solution was used for titration. All experiments were performed in four repetitions.

### 2.5. Statistical Analysis

The experimental results are presented as the mean ± standard deviation (SD). One-way analysis of variance (ANOVA) was performed and subsequently subjected to Duncan’s multiple range tests of the treatment mean by using Statistical Analysis System (SAS 9.4, SAS Institute, Cary, NC, USA), and the significance level was set at 0.05.

## 3. Results and Discussion

### 3.1. RF Heating Procedure Establishment for Lipase Inactivation

**Figure 1:**
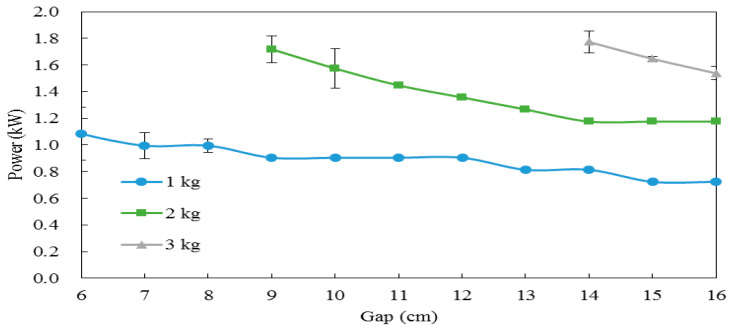
The radio frequency (RF) power output at different electrode gaps on 1, 2 and 3 kg rice bran packages. Data are expressed as the mean ± SD (*n* = 3).

As the electrode gap decreased, the RF power output increased under sample loading from 1 to 3 kg. For the same electrode gaps (14, 15 and 16 cm), a higher sample loading would have a higher RF power output (Figure 1). Besides, for power output stabilization and convenience of operating, a 1 kg rice bran loading was chosen for further experiments.

Table 1 indicate the heating rate and time from 25 °C to about 100 °C in the different electrode gaps (6, 8, 10 and 12 cm), and the results show that the highest heating rate (48.7 °C/min) was obtained in 6 cm, and the heating time was only 2 min. In addition, the energy consumption (0.15 kWh) was lower in this condition. For energy conservation and time efficiency, the electrode gap for 1 kg of rice bran loading was 6 cm, and it was suitable for heating rice bran from 25 °C to 100 °C in 2 min.

**Figure 2:**
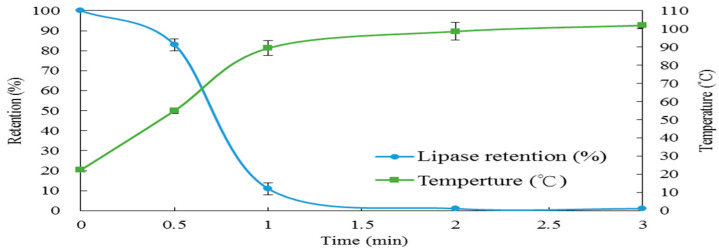
The temperature profile and lipase inactivation during RF for the 6 cm electrode gap with a 1 kg rice bran package. Data are expressed as the mean ± SD (*n* = 3).

Figure 2 illustrate the temperature profile and lipase activity retention (%) for the 6 cm electrode gap with 1 kg of vacuum-packed rice bran. The results showed the temperature reached above 70 °C, which is a lipase inactivation temperature, in 1 min. Besides, the lipase activity retention (%) was about 11%. When the time increased to 2 min, lipase activity retention (%) was close to zero, which indicate that RF could inactivate lipase efficiently and economically.

For microwave stabilization of the rice bran, the moisture content of the rice bran was adjusted to 21%, and then it was heated in an 800 W microwave convective dryer for 3 min and then dried in a hot air oven for a period of 1 h at 70 °C [24]. Rehydration could increase the loss factor of the 1 kg rice bran from about 14% to 21% in order to improve the temperature increasing rate during 3 min microwave heating, but it required to be hot-air dried for 1 h. However, using RF stabilization of rice bran did not require rehydration for improvement of the temperature increasing; therefore, RF saved the post-drying step to achieve a better efficiency than the microwave. Besides, Shi et al. [25] also worked on lipase inactivation of rice bran by RF treatment, and the temperature was set at 70 °C and 120 °C for 5 min, and the lipase activity retention (%) was 40% for 70 °C and 3% for 120 °C, respectively. It indicated lipase activity was not inactivated by a 70 °C RF treatment, and the reason might be that the electrode gap was not optimized, but the results also showed the lipase activity retention (%) was 3% under a 120 °C RF treatment. Similar results of lipase activity are shown in Figure 2. The temperature of the rice bran at 70 °C was not enough to completely inactivate lipase. However, when the temperature of the rice bran reached about 100 °C, it could completely inactivate lipase. Moreover, hot air (120 °C, 30 min), far-infrared (40 °C, 2000 W/m^2^, 2 h) and autoclaving (5 min) with hot air (50 °C, 3 h) were also studied for rice bran stabilization [8]. However, the sample loading of these studies was only 100 g, and the processing time was longer than that from our RF study.

In a previous study, 6 kW, 27.12 MHz RF for 200 g rice bran stabilization required 15 min [17], and 12 kW 27.12 MHz RF for 400 g rice bran stabilization required 6 min [19], respectively. Although the dielectric properties (ε′ and ε″) of rice bran at 27.12 MHZ were larger than 40.68 MHz [20], 1 kg rice bran at our 5 kW, 40.68 MHz RF heating system obtained a faster temperature increasing rate and less heating time (Table 1). Therefore, the rice loading and RF operation at different electrode gaps may influence the RF power output to obtain a better heating efficiency.

In our results, the original moisture content of rice bran was about 14% and did not need to be humidified to 21%. The 1 kg rice bran in the 5 kW, 40.68 MHz RF heating system required only 2 min, and has better potential for rice bran stabilization for food industry application, because it had no humidified pretreatment, a larger loading and a shorter treatment time.

### 3.2. Effect of RF Treatment on the Quality of Rice Bran

As shown in Table 2, the total phenolic and flavonoid contents of RF-treated rice bran were 0.61 mg gallic acid equivalent/g DW and 0.11 mg quercetin equivalent/g DW, respectively. There is no significant difference in total phenolic and flavonoid contents between the untreated and RF-treated rice bran. In addition, the DPPH free radical scavenging activity of the RF-treated rice bran was 84.8%, which was not significantly different from the untreated rice bran, but it was slightly lower than that of 5 mg/mL ascorbic acid (94.46%) and BHA (94.04%), respectively. Similar results were also found in the reducing power assay, and it reached 0.813 in RF-treated rice bran. These results indicate that the antioxidant components and activities of the RF-treated rice bran were still preserved after RF rapid heating processing. The protein, lipid and carbohydrate contents were 15.4%, 19.1% and 48.3% in the RF-treated rice bran. Therefore, RF-treated rice has potential as a nutritional supplement.

Wanyo et al. [8] investigated the changes in antioxidant activity and bioactive compounds in rice bran (100 g package, KDML 105 variety from northeastern Thailand) after hot-air, far-infrared radiation (FIR) and cellulase treatments, and they found the FIR-treated group (2000 W/m^2^, 40 °C 2 h) showed a higher DPPH radical scavenging activity (92.21%), ferric reducing antioxidant power (34.41 μmol FeSO_4_/g) and total phenolic content (4.05 mg gallic acid equivalent/g DW). A significant incensement of α-and γ-tocopherols also was found in the FIR-treated group. The antioxidant activity, total phenolic content and flavonoid content in the RF-stabilized rice bran were not significantly different from those in fresh rice bran (Table 1).

Shi et al. [25] showed that the unsuitable RF treatment for inactivating lipase would affect the quality of the rice bran. After RF at 120 °C for 5 min (2 kg package), the reducing sugar content of the rice bran was decreased, and it might be due to the amino-carboxyl reaction. Furthermore, similar results were obtained by Pradeep et al. [9], indicating that an improper high temperature would cause the loss of antioxidants. Rice bran (400 g) stabilized by the hot air-assisted RF heating system (12 kW, 27.12 MHz) at high temperatures of 110–115 °C for 6 min had no significant change in protein, lipid, total tocotrienol and total tocopherol content and fatty acid composition, only reducing the free phenolic content of the rice bran [19]. The RF heating at 100 to 120 °C holding for 5 min for stabilization of rice bran had a negative impact on the yield, purity, foaming properties and solubility of the rice bran protein isolates, but it improved the absorption and emulsifying properties [18]. The results showed that the antioxidant activity of rice bran could be preserved by the appropriate treatment, and a non-suitable heating treatment would affect the quality of the rice bran.

The L *, a * and b * values of the RF-treated rice bran were 16.13, 31.55 and 13.36, respectively (Table 3). There is no significant difference between the untreated and RF-treated rice bran, and it indicated the color was still stable after RF treatment (1 kg package, 6 cm electrode gap, from 25 °C to 100 °C in 2 min). The RF stabilization of the rice bran was operated at a high temperature and in a short time to avoid a color change. However, the color of the rice bran was darker after RF treatment at 120 °C for 5 min [25], and it might due to the high temperature that is causing a non-enzymatic, Millard browning reaction, and a change in the heat-sensitive components [4,26].

### 3.3. Storage Studies of Rice Bran Stabilized by RF Treatment

**Figure 3:**
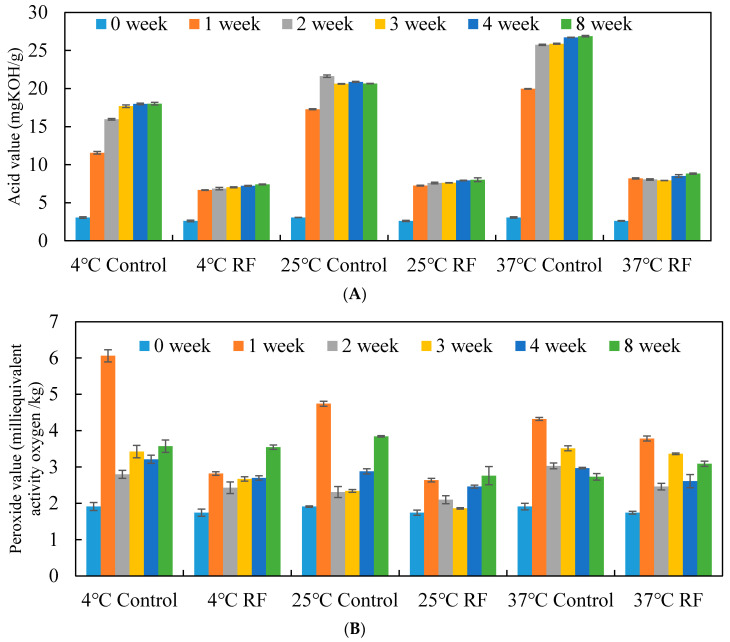

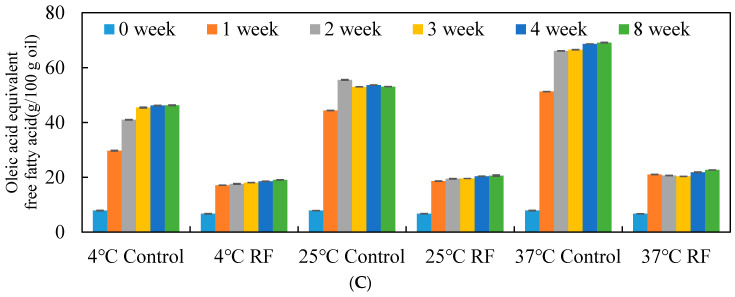
(**A**) The acid value, (**B**) free fatty acid content and (**C**) peroxide value change of RF-treated rice bran during different temperatures and storage periods. Data are expressed as the mean ± SD (*n* = 4).

As shown in Figure 3, the acid value of the RF-untreated rice bran (control group) was three times after 1 week at 4 °C storage, but the acid value of the RF-treated rice bran was still lower than that of the untreated (control) group at 4, 25 and, 37 °C. Besides, the acid value rose as the temperature increased, and the acid value of the untreated (control) group was obviously increased about 6.5 times at 37 °C. Although the acid value of the RF-treated group still increased about 2 times, the acid value remained steadily under 10 mg KOH/g after 8 weeks of storage. Furthermore, with the RF treatment, the free fatty acid contents were all below 20% after 8 weeks of storage, but the free fatty acid contents of the untreated (control) group was above 40%. Especially, the free fatty acid contents of the untreated (control) group after 8 weeks of storage at 37 °C reached 80%. The drastic accumulation of free fatty acid content in the untreated (control) group was in agreement with previous studies, founding that this could be accredited to the hydrolytic rancidity associated with lipase endogenously present in the rice bran [27,28].

In addition, similar results were observed in the evaluation of the peroxide value. The peroxide value is an indicator for the freshness of the lipid in the initial states of lipid oxidation by analyzing the content of the peroxides and hydro-peroxides. The acceptable limit of the peroxide value for rice bran is below 10 meg/kg according to the Codex Alimentations Commission. The peroxide value of the rice bran varied approximately from 6 to 3 meq/kg. The peroxide value of the RF-treated group was not only lower than that of the untreated (control) group, but it also remained well within the acceptable limit after 8 weeks of storage. The results indicate the efficacy of the RF treatment (1 kg package, 6 cm electrode gap, 2 min) in controlling the rise in acid value, free fatty acid and peroxide value, up to the end of an 8-week storage period at 4, 25 and 37 °C. Moreover, the DPPH free radical scavenging activity of the RF treatment group was maintained above 85% until 4 weeks, but it decreased to about 65% after 8 weeks of storage due to the long period of exposure to air (Table 4).

The acid value, free acid content and peroxide value of the untreated rice bran significantly increased after one week of storage at 4, 25 and 37 °C. Even if the rice bran was stored in a cold storage environment at 4 °C, it still could not slow down the lipase reaction. Once 1 kg of rice bran was stabilized by RF heating for only 2 min to inactivate lipase, the acid values and free fatty acids of the rice bran were not significantly different during storage in the following 1 to 8 weeks, regardless of storage at 4, 25 or 37 °C (Figure 3). Similar results showed that RF-stabilized rice bran decrease the lipase and lipoxygenase activities; therefore, the free fatty acid content and peroxide value of the extracted RB oil remained below the acceptable limits even following 60 days of storage at 35 °C [17]. The free fatty acid content of fresh rice bran increased from 10 to above 30% after 15 days and 60% after 30 days of storage, but the infrared-stabilized rice bran had below 10% free fatty acid [29]. The rice bran could be stabilized by using microwaves without any loss of nutrition quality during three months of storage [28]. However, for the bulk product, the advantage of RF treatment over middle infrared and microwave is a deeper penetration depth and uniform heating effect [16].

## 4. Conclusions

When the RF electrode gap was controlled at 6 cm, it only took 2 min to heat the 1 kg rice bran package from 20 °C to 100 °C, and the temperature distribution was uniform. In addition, the lipase was completely inactivated, and the quality still remained in good condition. The change in color was not significant after RF treatment, and the RF treatment would not affect the antioxidant activity. The RF-treated rice bran could be stored safely for a period of 8 weeks at 4, 25 and 37 °C, without rancidity. The acid value, free fatty acid content and peroxide value of the RF-treated rice bran met the quality standard. In summary, this work provides valuable information (1) to establish the radio frequency (RF) heating procedure for inactivating the lipase in rice bran; and (2) to understand the effect of RF treatment on lipase activity, antioxidant activity, color and quality during storage. Furthermore, this study shows the great potential of RF technology for stabilizing rice bran.

## Figures and Tables

**Table 1:** The temperature profile equation, heating rate, heating time and energy consumption of rice bran by RF treatment.

Gap (cm)	Temperature Profile	R^2^	Heating Rate (°C/min)	Heating Time (min)	Energy Consumption (kWh)
6	y = 48.724x + 25.50	0.999	48.7	1.5	0.15
8	y = 36.032x + 23.14	0.985	36.0	2	0.20
10	y = 27.466x + 20.10	0.979	27.5	3	0.31
12	y = 22.746x + 19.33	0.961	22.7	4	0.41

Data are expressed as the mean (*n* = 3). Heating time: from 25 °C to 100 °C

**Table 2:** The antioxidant components and activities analyses of the RF-treated rice bran.

Sample	Total Phenolic Content (mg Gallic Acid Equivalent/g)	Flavonoid Content (mg Quercetin Equivalent/g)	Scavenging DPPH Free Radicals (%)	Reducing Power
Untreated	0.63 ± 0.19 ^a^	0.12 ± 0.01 ^a^	86.80 ± 1.38 ^b^	0.813 ± 0.08 ^b^
RF treated	0.61 ± 0.23 ^a^	0.11 ± 0.03 ^a^	84.80 ± 2.59 ^b^	0.805 ± 0.03 ^b^
Ascorbic acid	-	-	93.46 ± 0.63 ^a^	1.08 ± 0.01 ^a^
BHA	-	-	94.04 ± 0.00 ^a^	0.59 ± 0.01 ^c^

Data are expressed as the mean ± SD (*n* = 4). ^a–c^ Means with a different superscript in the same column were significantly different (*p* < 0.05). The concentration of rice bran was 50 mg/mL. The concentration of ascorbic acid and BHA were 5 mg/mL.

**Table 3:** Color analyses of the RF-stabilized rice bran.

Rice Bran	L *	a *	b *
Original	16.16 ± 0.15	31.50 ± 0.17	13.40 ± 0.23
RF stabilization	16.13 ± 0.13	31.55 ± 0.21	13.36 ± 0.26

Data are expressed as the mean ± SD (*n* = 6). Means in the same column were not significantly different (*p* > 0.05).

**Table 4:** Changes in the DPPH free radical scavenging ability of the RF-treated rice bran during different temperature storage.

Time (Week)	Temperature (°C)	Rice Bran	Scavenging DPPH Free Radicals (%)
0	-	Control	88.12 ± 0.01
	-	RF	88.34 ± 0.01
4	4	Control	86.73 ± 0.07
		RF	84.78 ± 0.06
	25	Control	86.53 ± 0.04
		RF	85.93 ± 0.05
	37	Control	87.31 ± 0.11
		RF	85.90 ± 0.08
8	4	Control	67.36 ± 0.23
		RF	65.13 ± 0.42
	25	Control	68.90 ± 0.06
		RF	65.27 ± 0.19
	37	Control	72.52 ± 0.40
		RF	65.48 ± 0.35

Data are expressed as the mean ± SD (*n* = 4).
